# Plantar Pressure Variations Induced by Experimental Malocclusion—A Pilot Case Series Study

**DOI:** 10.3390/healthcare9050599

**Published:** 2021-05-18

**Authors:** Simona Maria Iacob, Andrea Maria Chisnoiu, Smaranda Dana Buduru, Antonela Berar, Mirela Ioana Fluerasu, Ioana Iacob, Adriana Objelean, Wilhelm Studnicska, Liviu Marin Viman

**Affiliations:** 1 Prosthodontics Department, Faculty of Dental Medicine, “Iuliu Hatieganu” University of Medicine and Pharmacy, 400006 Cluj-Napoca, Romania; simona72cj@yahoo.com (S.M.I.); antonela_berar@yahoo.com (A.B.); fluerasu.mirela@umfcluj.ro (M.I.F.); 2 Faculty of General Medicine, “Iuliu Hațieganu” University of Medicine and Pharmacy, 6, Victor Babes Street, 400008 Cluj-Napoca, Romania; iacob.ioana123@yahoo.com; 3 Department of Dental Materials and Ergonomics, Faculty of Dental Medicine, “Iuliu Hatieganu” University of Medicine and Pharmacy, 400083 Cluj-Napoca, Romania; adriana.caracostea@umfcluj.ro; 4 Faculty of General Medicine, “Iuliu Hațieganu” University of Medicine and Pharmacy, 400008 Cluj-Napoca, Romania; wilhelm.studnicska@yahoo.com; 5 Applied Electronics Department, Faculty of Electronics, Telecommunications and Information Technology, Technical University of Cluj-Napoca, 400027 Cluj-Napoca, Romania; liviu.viman@ael.utcluj.ro

**Keywords:** dental occlusion, posture, plantar pressure, baropodometric analysis, malocclusion

## Abstract

Background: All body systems involved in ensuring a healthy posture (musculoskeletal system, oculomotor, oto-vestibular and occluso-cranial-mandibular) are essential in maintaining postural balance. Research Question: Does experimental malocclusion in subjects in static position determine variations in plantar pressure? Methods: Overall, 31 subjects were included in the study. The plantar pressure was evaluated in five different points: lateral and medial heel, midfoot, 1st and 5th metatarsal area. Using a specially designed splint, an artificial malocclusion was induced on the right hemimandibular arch. The pressure was measured at 0 (T0), 15 (T1) and 30 min (T2) after splint application. Results: The right external plantar sensors recorded statistically significant differences in pressure values after 15 min of splint wear (5th metatarsal area, *p* = 0.05; midfoot, *p* = 0.04). Important pressure values were also recorded by the left internal plantar sensors (1st metatarsal, *p* = 0.01; medial heel, *p* = 0.006), after 30 min of splint wear. Conclusions: Asymmetrical experimental malocclusion produces early changes in plantar pressure, a proof of compensatory mechanisms induced by secondary postural imbalance.

## 1. Introduction

Foot posture is determined by the integration of sensitive information (stimuli) received from the peripheral receptors of the organism [1]. Correct biomechanics of the foot maintain a good posture and an even pressure distribution on the plantar surface [2]. Postural control is maintained by continuous equilibrated contraction of anti-gravitational body musculature. Alterations in the position of the body may lead to variations of load due to the acceleration of gravity and influence the plantar pressure. Several methods have been used for posture analysis, such as electromyography (EMG), Romberg test analysis, pressure platform analysis, and thermography [3,4,5].

Studies present contradictory results regarding the influence of malocclusion in posture balance. Several studies suggest that spatial relationships between the jaws may influence the distal musculature and induce body postural adaptations [6,7]. However, Perinetti et al. concluded that mandibular position, asymmetric occlusion, and temporomandibular disorders do not appear to correlate with body sway or muscle activity in other parts of the body, including those responsible for maintaining posture, at a clinically relevant level [8,9].

The posture of the foot can cause orthostatic imbalances or may cause pathologic modifications in other parts of the body (oculomotor system, stomatognathic system, etc.) [6]. Martinez et al. consider that determining the plantar pressure using baropodometric measurements represents an important element in understanding the physiopathology of postural anomalies and the diagnostic process [7].

Bricot states that postural modifications may have either an upward progression with a plantar starting point or a downward path beginning from the head (eyes, temporomandibular joints, vestibular system) [6]. A dental occlusion disturbance stimulates the trigeminal nerve, which induces a muscular and articular chain reaction. Anatomical connections of the trigeminal nerve with the cervical spine, in continuity with the lumbar spine that participates in plantar sensibility, can determine the static postural symptoms [10].

Baropodometric analysis conducted using plantar pressure platforms may reveal certain dysfunctions in the foot, regardless of the cause. The analysis generates a plantar map with various pressure points caused by a pathological posture [11]. The method creates an image of the foot together with the pressure exerted on the ground when the body is in orthostatic posture. Thus, the symmetry of both soles and the pressure in different areas of the sole can be measured. The weight distribution of the body on both feet can be expressed in percentages. Not only can this method help verify the orthostatic posture, but also analyze the way it can be altered by certain pathologies. The orthostatic posture can be evaluated using this method, at the same time establishing the way it can be altered by certain pathologies [6,10,12].

Due to their implication in the development and evolution of major postural changes, it is mandatory to treat dental malocclusions [13]. The temporomandibular disorder (TMD) is a disease that affects temporomandibular joints (TMJs), masticatory muscles, and neighboring anatomical parts [14]. It is known that a lower jaw position can affect body movement and that occlusal stability has a positive effect on static and dynamic posture [15,16].

Some authors state that all the systems which help maintain a healthy posture (the musculoskeletal, oculomotor, oto-vestibular and occluso-cranio-mandibular systems) are essential in maintaining postural balance [6,11,17].

The aim of the current study is to evaluate how the plantar pressure in static position changes in subjects whose occlusion had been temporarily modified into malocclusions.

## 2. Materials and Methods

This is an analytical, observational, prospective, transverse and cohort study that was conducted in the Prosthodontics Department of the “Iuliu Hațieganu” University of Medicine and Pharmacy Cluj-Napoca, from April 2018 to December 2018. The Ethical Commission of the University approved the experimental methodology (agreement no. 156/02.04.2018).

In total, 31 volunteers, all students attending the Faculty of Medicine, were included in the study. These participants were all young, Caucasian, healthy adults with normal occlusion and Angle’s class I (based on intermolar relationship). The mean age of the volunteers was 23.77 years, with the youngest being 19 years of age and the oldest 46. The standard deviation was 6.402. Of the 31 subjects, 18 were females (58.1%) and 13 were males (41.9%). The sample size was relatively small, so it was considered a preliminary study.

The including criteria were as follows: good general health (lack of medical, psychosocial or emotional conditions), no prior accidents or surgery which could affect the posture, absence of visual and auditory dysfunctions, absence of joint diseases that could affect the posture, absence of any previously diagnosed posture pathology, the presence of all 28 natural teeth, absence of dentomaxillary anomalies, absence of extended dental treatments, absence of craniomandibular or temporomandibular disorders (according to Research Diagnosis Criteria for Temporomandibular Joint Dysfunction) [18], and no ongoing orthodontic treatment.

The clinical occlusal examination for each subject was conducted by the same doctor, with 20 years of experience in the field, using the same protocol. The pedometer is a device specifically designed to measure plantar pressure at multiple, precisely defined points. The current study evaluates the possibility of a connection between occluso-dental dysfunctions and posture pathology by measuring the pressure of five specific points, for both feet: lateral heel (external side of the heel), medial heel (internal side of the heel), midfoot (middle of the plantar surface), 1st metatarsal area and 5th metatarsal area.

The plantar pressure was evaluated at different times, using an experimental pedometer designed by the Cluj-Napoca Technical University Department of Applied Electronics. The pedometer is a device specifically designed, validated and calibrated to measure plantar pressure at multiple, precisely defined points. The current study evaluates the possibility of a connection between occluso-dental dysfunctions and posture pathology by measuring the pressure of five specific points, for both feet: lateral heel (external side of the heel), medial heel (internal side of the heel), midfoot (middle of the plantar surface), 1st metatarsal area and 5th metatarsal area.

Shu et al. [19] mentioned that the sole of foot could be divided into 4 major areas, each of them including several subareas: heel (area 1–3), midfoot (area 4–5), metatarsal (area 6–10) and toe (area 11–15). These areas support most of the body weight and are adjusted by the body’s balance; therefore, we included 5 sensors in the areas where most pressure occurs during static position.

The components of the pedometer are:

1. The platform supporting the measuring components: 10 FSR (force-sensing resistor) sensors for measuring the pressure force in the foot, 5 sensors for each foot.
2. cRIO-9068 controller (the central component of the system): a chassis created by National Instruments which can be used in many areas of work as it can be connected to many devices. It can be connected to the PC through the Ethernet port and serial ports RS-232, RS-485. This is an embedded real-time controller, with the possibility of FPGA reconfiguration for C series modules. It has an ARM Cortex-A9, 667 MHz dual-core processor, 2 Ethernet ports, serial ports RS-232 and RS-485, 8 slots for module connection, 1 GB non-volatile memory (Hard Drive) and 512 MB Ram. It requires a power source of 9–30 V with a maximum power of 25 W. It supports the LabView FPGA graphic software.
3. The NI 9219 universal C Series module used for sensor measurement. With the NI 9219, we can measure several signals from sensors such as strain gages, RTDs, thermocouples and other powered sensors. The channels are individually selectable, so we can perform a different measurement type on each of the four channels.
4. User interface (LabView FPG graphic software located on the PC): for data collection and analysis.

The subjects were informed about the methodology, duration and the implications of this experiment. The study was anonymous, and all subject expressed their consent.

### Method of Examination

**Figure 1:**
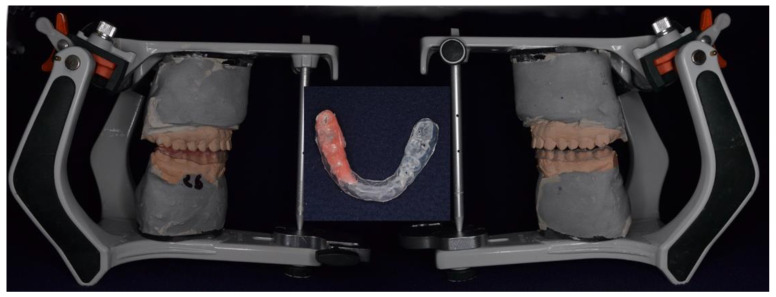
Cast mounted in semi-adjustable articulator and rigid mandibular splint design.

After oral and TMJ examination, each patient had alginate impressions of both arches taken. The resulting casts were mounted in maximum intercuspation (MI) in a semi-adjustable articulator Bio Art, A7 plus (Bio-Art, Brazil). A full-coverage rigid mandibular splint was made. Adding self-curing resin on the right side created an occlusal imbalance and the bite was raised unilaterally. The incisal pin of the articulator was therefore raised by 2 mm from the MI (Figure 1). This way, an experimental occlusal imbalance was created. With the help of the plantar platform, three 30-s-long podometric measurements were recorded for each subject. The measurements were made as follows: the first one was recorded before applying the splint (with the lower jaw in rest position, having no dental contacts) with the eyes open; the second measurement was recorded 15 min after applying the occlusal device and the third one 30 min after applying the splint. The value of the measurement in each moment (T0, T1, T2) was obtained by calculating the mean of 10 consecutive registrations at 3-s intervals.

Silence was maintained during the examination and all factors that could cause unrest in the subjects were removed. The participants were asked to sit relaxed in orthostatic position, with their arms alongside their body, gazing at the horizon. They were prohibited to consume alcohol or practice sports 24 h prior to the examination. After applying the splint in the mouth, each patient was asked to bite and maintain tooth contact on the splint for 30 min, without excessive force until the completion of the measurement. The pressure exerted on each well-defined point on the plantar surface was measured in three moments: T0, T1 (15 min) and T2 (30 min) of splint wearing. Then, 10 sensors were used to measure the force applied on each point, 5 sensors on each sole. The data were expressed in g/cm^2^ and statistically analyzed.

The statistical analysis was conducted using MedCalc Statistical Software version 18.11 (MedCalc Software bvba, Ostend, Belgium; http://www.medcalc.org; 2018 (accessed on 1 March 2021)) The data were analyzed using the Kolmogorov–Smirnov test for normal distribution and was expressed through median and percentiles 25 and 75. The comparison between the measured values was carried out using the Wicoxon test (*p* ≤ 0.05).

## 3. Results

**Figure 2:**
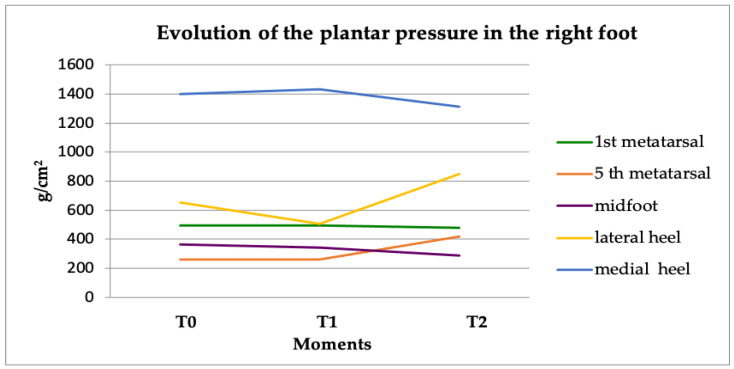
Evolution of plantar pressure in the right foot.

Data analysis revealed an increase in pressure after splint application on the right foot, in the 5th metatarsal bone area as well as in the lateral heel surface (Figure 2).

Statistical analysis revealed that the sensor for the 5th metatarsal area (*p* = 0.05) together with the one corresponding to the midfoot plantar surface (*p* = 0.04) recorded significant values at 15 min after applying the oral splint on the right side (Table 1).

The increased pressure recorded in the lateral area of the right heel and 5th metatarsal bone after inducing the occlusal imbalance on the same side emphasizes the rapid response reflected on the body posture. The body tilts to the same side, in this case the right side, which explains the increased pressure values measured by the external sensors of the right foot.

**Figure 3:**
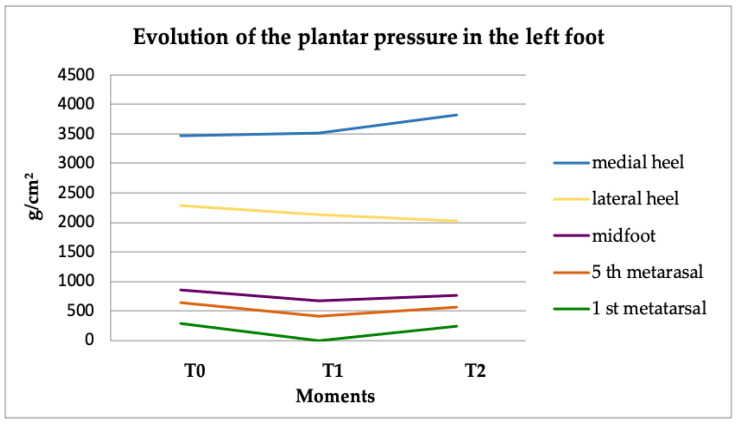
Evolution of plantar pressure in the left foot.

The left foot underwent the same analysis: as the patient wore the splint, a decrease in pressure levels was recorded in the 5th metatarsal area and in the lateral heel along with an increase in pressure levels in the medial heel (Figure 3).

Statistically significant data were found for the variations of pressure values at the internal side of the left foot: left 1st metatarsal area (*p* = 0.01) and medial left heel (*p* = 0.006) at T2 (Table 2).

The sensors for the medial left heel and the left 1st metatarsal area recorded an increase in pressure values at T1 and T2.

The results of the measurements in the left foot also suggest that the increased pressure was caused by the tilting of the body to the right side, the same side where the occlusal imbalance was induced.

When comparing the podal pressure modifications from the left and the right side, a significant difference was observed in the heel areas at T1 (*p* < 0.005).

## 4. Discussion

In a correct posture, the line of gravity passes through the axes of all joints of the vertically aligned body segments. It is important for the head, trunk, shoulders and pelvic girdle to be in mechanical and muscular balance [20].

It is possible to assess the body posture through different clinical examinations with the help of a chiropractor or a posturologist. Clinical examination of the body posture is time-consuming and requires in-depth knowledge. Paraclinical examinations are also useful, an example being the use of a podometric platform in measuring the pressure applied by the foot.

The current study evaluated the possible link between artificially induced dental malocclusion and variations of plantar pressure. The method used in this study is original since the maloccusion was induced using individually designed splints, which are more stable and precise compared to cotton rolls used in previous research [21,22]. The results revealed that dental malocclusion can cause significant modifications in sole pressure.

Similar results were obtained by Budd et al., who investigated to what degree occlusal imbalance, or the correction of dental occlusion, affects the force distribution through the whole body, as well as the sport performance of professional athletes. The researchers applied cotton rolls between the superior and inferior premolars of each participant of the two groups of athletes in order to create or treat a dental occlusion imbalance, depending on the case. In the majority of cases (85%), computer-generated images of sole pressure revealed a direct link between dental occlusion and sole pressure. The study showed that athletes who had a stable occlusion also had better sport performances compared to those with dental malocclusions [21].

The study conducted by Cuccia showed that occlusal overload causes a direct increase in sole pressure. The results revealed that modifying the sole pressure distribution in the anterior and posterior parts of the foot by applying cotton rolls between the opposing teeth to create a dental malocclusion may influence body posture [22]. This is because the mechanoreceptors of the tendons of the muscles governing the plantar arch configuration are stimulated in different ways during the activation of long osteo-arthro-muscular chains [23].

These posture modifications can be attributed to asymmetrical orofacial muscle contraction. This imbalance is transmitted to the brain which may then, via the trigeminal nerve, cause asymmetrical hypertonicity of the neck muscles that is further propagated downwards along the spine to the limbs.

Opposing results were obtained by Ferrario et al., who could not find any connection between plantar pressure distribution and TMD or dental malocclusion [24].

A significant association between postural changes and altered muscle function was observed by Nicolakis et al. in patients with TMDs, when compared to healthy individuals. These postural abnormalities included changes in the cervical region, increased thoracic kyphosis and lumbar lordosis, protruding abdomen and shoulder abduction [25].

The lower jaw affects the head position and can influence body posture. Proprioceptors of the mandible, alongside the trigeminal nerve, masticatory muscles and periodontal ligaments [26] contribute to the control of the head posture via the sternocleidomastoid muscle [27]. Sakaguchi et al. performed a digital analysis of dental occlusion forces on a group of 45 asymptomatic individuals. The study evaluated the effect of changing the mandibular position relative to the body posture, and vice versa, by using a force platform. Statistically significant differences were observed in the sway length and sway area parameters between different mandibular positions, and the occlusal load values showed a significant difference when a heel lift was positioned under the right foot. It was concluded that there is a direct link between the mandibular position and the body posture [28].

A recent study performed by Amaricai et al. established a strong correlation between different dental occlusion conditions and plantar pressure. In this study, plantar pressure was assessed and stabilometric analysis was performed in mandibular postural position, maximum intercuspation, biting on cotton rolls and maximum mouth opening. The results showed that, in young adults with an optimum functional occlusion, the maximum mouth opening influences the static plantar pressure. Improved postural stability was recorded in maximum intercuspation compared to the rest of the positions analyzed in the study [29].

These findings are similar to the ones obtained in the current study, with results showing increased plantar pressure on the homolateral side with the induced malocclusion.

Similar results were obtained by La Touche et al., who concluded that the experimental induction of different craniocervical postures influences the maximal mouth opening and pressure pain threshold values of the temporomandibular joint as well as the muscles of mastication that receive motor and sensory innervation via the trigeminal nerve. The influence of craniocervical posture on the maximal mouth opening and pressure pain threshold was found in patients with myofascial temporomandibular pain disorders [30].

Opposing results have been reported by Scharnweber et al., who concluded that persistent dental parameters have no effect on postural sway, postural control or plantar pressure distribution, being found to be an independent postural criterion [31]. Similar results were obtained by De Giorgi et al. [32].

The present study presents several limitations: namely, the reduced sample size and the lack of a control group. Another aspect to take into consideration for further studies is the possible correlation with the arch shape of the foot. Nonetheless, the obtained results are encouraging, and the current study contained adequate measurement properties for statistical analysis. The methodology introduced in the present study could also supply information on the influence of nonfunctional dental occlusion, as it consisted of evaluations regarding pressure in different podal segments. With the development of a valid and reliable methodology, investigations to establish the normal range of podal pressure in different populations, ethnic groups and skeletal types can be performed. The development of a valid and reliable methodology for measuring podal pressure will also facilitate investigations regarding the relationship between dental occlusion, posture and TMDs, as well as other human postural disorders.

The current study evaluated the modifications of podal pressure in orthostatic position. Dynamic studies are necessary and orofacial muscle contraction monitorization should be included. This is because as they are walking, teeth contacts vary in intensity and number in subjects with dental malocclusion as opposed to those with a healthy occlusion. This may cause an imbalance in pressure distribution to the sole while walking. The authors consider it important to monitor the posture during complex oral rehabilitation, due to the modifications that could appear.

## 5. Conclusions

All the patients included in this study had modifications in plantar pressure when the artificial malocclusion was induced, indicating that dental occlusion may represent an important etiologic factor in postural imbalance. Significant differences in plantar pressure were recorded on the external sensors of the ipsilateral foot and the internal sensors of the contralateral foot with increased values compared to the initial moment, whereas the external sensors of the contralateral foot recorded a decrease in pressure values.

## Figures and Tables

**Table 1:** The values of pressure recorded from the sole in the right foot (the same side as the occlusal imbalance).

Pressure Value (g/cm^2^)	T0 (0 min)	T1 (15 min)	T2 (30 min)	*p* 0–15	*p* 0–30
Right 1st metatarsal area	497.2 (189.2; 839.1)	496.9 (307.6; 740.7)	480.8 (275.4; 862.4)	0.2	0.2
Right 5th metatarsal area	259.8 (219.6; 692.2)	260.5 (188.5; 702)	416.7 (170.6; 671.8)	0.05 *	0.2
Right midfoot	361.4 (95; 628.3)	339.8 (90.7; 488.4)	288.3 (100.4; 661.9)	0.04 *	0.6
Right lateral heel	655.2 (280.8; 1260.6)	504.8 (259.4; 1200.1)	851 (462.6; 1325.1)	0.5	0.3
Right medial heel	1397.9 (883.5; 1717.1)	1434.4 (879.9; 2426.4)	1311.4 (417.3; 2541.3)	0.2	0.5

* Statistically significant (*p* ≤ 0.05).

**Table 2:** The values of pressure recorded from the sole in the left foot (opposite side as the occlusal imbalance).

Pressure Value (g/cm^2^)	T0 (0 min)	T1 (15 min)	T2 (30 min)	*P* 0–15	*P* 0–30
Left 1st metatarsal area	286 (75.9; 441.6)	253.4 (112.9; 733.1)	247.2 (118.6; 838.3)	0.1	0.01 *
Left 5th metatarsal area	354 (141; 553.2)	416.2 (154.4; 628.3)	319.2 (179.8; 685.1)	0.09	0.6
Left midfoot	215.7 (88.5; 367.1)	248.7 (53.3; 406.7)	198.8 (47; 446.3)	0.5	0.1
Left lateral heel	1427.7 (876.5; 2088.5)	1461.2 (707.5; 2084.1)	1260.5 (709.9; 2117.5)	0.6	0.4
Left medial heel	1178.1 (593.2; 1783.4)	1386.7 (593.2; 2216)	1795.5 (845.1; 2487.3)	0.09	0.006 *

* Statistically significant (*p* ≤ 0.05).

## Data Availability

Data supporting the reported results can be found by contacting the corresponding authors.

## References

[1] Neto H.P., Grecco L.A., Braun Ferreira L.A. (2015). Clinical analysis and baropodometric evaluation in diagnosis of abnormal foot posture: A clinical trial. J. Bodyw. Mov. Ther..

[2] Vianna D.L., Greve J.M.D. (2006). Relação entre a mobilidade do tornozelo e pé e a magnitude da força vertical de reação do solo [Relationship between ankle and foot mobility and the magnitude of the vertical ground reaction force). Rev. Bras. Fisioter..

[3] Rodríguez-Sanz D., Becerro-de-Bengoa-Vallejo R., López-López D., Calvo-Lobo C., Martínez-Jiménez E.M., Perez-Boal E., Losa-Iglesias M.E., Palomo-López P. (2018). Slow velocity of the center of pressure and high heel pressures may increase the risk of Sever’s disease: A case-control study. BMC Pediatr..

[4] Martínez-Jiménez E.M., Losa-Iglesias M.E., Díaz-Velázquez J.I., Becerro-De-Bengoa-Vallejo R., Palomo-López P., Calvo-Lobo C., López-López D., Rodríguez-Sanz D. (2019). Acute Effects of Intermittent Versus Continuous Bilateral Ankle Plantar Flexor Static Stretching on Postural Sway and Plantar Pressures: A Randomized Clinical Trial. J. Clin. Med..

[5] Martínez-Jiménez E.M., Losa-Iglesias M.E., Antolín-Gil M.S., López-López D., Romero-Morales C., Benito-de-Pedro M., Calvo-Lobo C., Becerro-de-Bengoa-Vallejo R. (2021). Flexor Digitorum Brevis Muscle Dry Needling Changes Surface and Plantar Pressures: A Pre-Post Study. Life.

[6] Bricot B. (2020). La Reprogrammation Posturale Globale (Global Postural Reprogramming).

[7] Martinez-Nova A., Sanchez-Rodriguez R., Cuevas-Garcia J.C. (2007). Estudio baropodométrico de los valores de presión plantar en pies no patológicos (Baropodometry study of plantar pressure values in normal feet). Rehabilitación.

[8] Perinetti G., Turp J.C., Primožič J. (2011). Associations between the masticatory system and muscle activity of other body districts. A meta-analysis of surface electromyography studies. J. Electromyogr. Kinesiol..

[9] Perinetti G., Primožič J., Manfredini D. (2013). The diagnostic potential of static body-sway recording in orthodontics: A systematic review. Eur. J. Orthod..

[10] Clauzade M. (2015). L’occlusion dentaire. Rev. Orthod. Clinique..

[11] Bellizzi M., Rizzo G., Bellizzi G. (2011). Electronic baropodometry in patients affected by ocular torticollis. Strabismus.

[12] Kaercher C.W., Genro V.K., Souza C.A. (2011). Baropodometry on women suffering from chronic pelvic pain—A cross-sectional study. BMC Women’s Health.

[13] Milani R.S., de Perière D.D., Lapeyre L. (2000). Relationship between dental occlusion and posture. Cranio.

[14] Okeson J.P. (2012). Management of Temporomandibular Disorders and Occlusion.

[15] Fujimoto M., Hayakawa I., Hirano S. (2001). Changes in gait stability induced by alteration of mandibular position. J. Med. Dent. Sci..

[16] Okubo M., Fujinami Y., Minakuchi S. (2010). The effect of complete dentures on body balance during standing and walking in elderly people. J. Prosthodont. Res..

[17] Maeda N., Sakaguchi K., Mehta N.R. (2011). Effects of experimental leg length discrepancies on body posture and dental occlusion. Cranio.

[18] Schiffman E., Ohrbach R., Truelove E. (2014). Diagnostic criteria for temporomandibular disorders (DC/TMD) for clinical and research applications: Recommendations of the International RDC/TMD Consortium Network and Orofacial Pain Special Interest Group. J. Oral. Facial. Pain. Headache..

[19] Shu L., Hua T., Wang Y., Li Q., Feng D.D., Tao X. (2010). In-Shoe Plantar Pressure Measurement and Analysis System Based on Fabric Pressure Sensing Array. Trans. Inf. Tech. Biomed..

[20] Fernández R.F., Carter P., Muñoz S. (2016). Evaluation of validity and reliability of a methodology for measuring human postural attitude and its relation to temporomandibular joint disorders. Singapore Med. J..

[21] Budd S.C., Egea C.J. (2017). Dental occlusion and athletic performance. Sport and Oral Health. A Concise Guide.

[22] Cuccia A.M. (2011). Interrelationships between dental occlusion and plantar arch. J. Bodyw. Mov. Ther..

[23] Valentino B., Ferrara I., Valentino T., Paparo E., di Giacinto S., Valentino R. (2005). An innovative electromyographic test for use in the early diagnosis of scoliosis in school-age children. Pain. Clinic..

[24] Ferrario V.F., Sforza C., Schmitz J.H. (1996). Occlusion and center of foot pressure variation: Is there a relationship?. J. Prosthet. Dent..

[25] Nicolakis P., Nicolakis M., Piehslinger E. (2000). Relationship between craniomandibular disorders and poor posture. Cranio.

[26] Huggare J. (1998). Postural disorders and dentofacial morphology. Acta Odontol. Scand..

[27] Kibana Y., Ishijima T., Hirai T. (2002). Occlusal support and head posture. J. Oral. Rehabil..

[28] Sakaguchi K., Mehta N.R., Abdallah E.F. (2007). Examination of the relationship between mandibular position and body posture. Cranio.

[29] Amaricai E., Onofrei R.P., Suciu O. (2020). Do different dental conditions influence the static plantar pressure and stabilometry in young adults?. PLoS ONE.

[30] La Touche R., París-Alemany A., von Piekartz H. (2011). The influence of cranio-cervical posture on maximal mouth opening and pressure pain threshold in patients with myofascial temporomandibular pain disorders. Clin. J. Pain..

[31] Scharnweber B., Adjami F., Schuster G. (2017). Influence of dental occlusion on postural control and plantar pressure distribution. Cranio.

[32] De Giorgi I., Castroflorio T., Cugliari G. (2018). Does occlusal splint affect posture? A randomized controlled trial. Cranio.

